# Per- and polyfluoroalkyl substances (PFAS) and thyroid hormone measurements in dried blood spots and neonatal characteristics: a pilot study

**DOI:** 10.1038/s41370-023-00603-4

**Published:** 2023-09-20

**Authors:** Ana K. Rosen Vollmar, Elizabeth Z. Lin, Sara L. Nason, Katerina Santiago, Caroline H. Johnson, Xiaomei Ma, Krystal J. Godri Pollitt, Nicole C. Deziel

**Affiliations:** 1 Department of Environmental Health Sciences, Yale School of Public Health, New Haven, CT USA; 2 Departments of Environmental Science and Forestry and Analytical Chemistry, Connecticut Agricultural Experiment Station, New Haven, CT USA; 3 Department of Chronic Disease Epidemiology, Yale School of Public Health, New Haven, CT USA

**Keywords:** PFAS, Per- and polyfluoroalkyl substances, Dried blood spot, Thyroid hormone, Newborn, Environmental exposure

## Abstract

**Background:**

Pediatric thyroid diseases have been increasing in recent years. Environmental risk factors such as exposures to chemical contaminants may play a role but are largely unexplored. Archived neonatal dried blood spots (DBS) offer an innovative approach to investigate environmental exposures and effects.

**Objective:**

In this pilot study, we applied a new method for quantifying per- and polyfluoroalkyl substances (PFAS) to 18 archived DBS from babies born in California from 1985–2018 and acquired thyroid hormone measurements from newborn screening tests. Leveraging these novel data, we evaluated (1) changes in the concentrations of eight PFAS over time and (2) the relationship between PFAS concentrations, thyroid hormone concentrations, and neonatal characteristics to inform future research.

**Methods:**

PFAS concentrations in DBS were measured using ultra-high-performance liquid chromatography-mass spectrometry. Summary statistics and non-parametric Wilcoxon rank-sum and Kruskal–Wallis tests were used to evaluate temporal changes in PFAS concentrations and relationships between PFAS concentrations, thyroid hormone concentrations, and neonatal characteristics.

**Results:**

The concentration and detection frequencies of several PFAS (PFOA, PFOS, and PFOSA) declined over the assessment period. We observed that the timing of specimen collection in hours after birth was related to thyroid hormone but not PFAS concentrations, and that thyroid hormones were related to some PFAS concentrations (PFOA and PFOS).

**Impact statement:**

This pilot study examines the relationship between concentrations of eight per- and polyfluoroalkyl substances (PFAS), thyroid hormone levels, and neonatal characteristics in newborn dried blood spots (DBS) collected over a period of 33 years. To our knowledge, 6 of the 22 PFAS we attempted to measure have not been quantified previously in neonatal DBS, and this is the first study to examine both PFAS and thyroid hormone concentrations using DBS. This research demonstrates the feasibility of using newborn DBS for quantifying PFAS exposures in population-based studies, highlights methodological considerations in the use of thyroid hormone data for future studies using newborn DBS, and indicates potential relationships between PFAS concentrations and thyroid hormones for follow-up in future research.

## Introduction

Per- and polyfluoroalkyl substances (PFAS) are a family of synthetic chemicals with thyroid hormone-disrupting properties [1]. PFAS are heat-resistant, chemically stable, and repel stains, properties which led to their widespread inclusion in commercial and industrial products. PFAS have been widely detected in drinking water [2, 3], measured in raw, processed, and packaged foods and animal products [4], and are found in cookware, food packaging materials, household products, textiles, clothing, lubricants, and firefighting foams [5]. PFAS originating from indoor sources can accumulate in settled dust [6, 7]. Because PFAS are highly resistant to degradation and remediation, they can remain in the human body and the environment for extended periods of time [8, 9].

Studies in United States (US) populations have detected one or more PFAS in nearly all blood samples collected from pregnant women and children [10–12]. PFAS readily cross the placenta and are detected in umbilical cord blood, with correlations between maternal serum and cord blood concentrations, underscoring the importance of the in utero exposure pathway [13–17]. Postnatally, children can be exposed through drinking water and dietary sources, incidental ingestion of house dust due to their propensity to engage in hand-to-mouth activity, and inhalation of PFAS-laden dust [5, 6, 18]. Prenatal and postnatal exposures exhibit moderate correlations that vary in magnitude depending on the chemical and age of the child [19].

PFAS exposure has been linked to various health endpoints, including dyslipidemia in adults and adverse immunosuppressive and developmental effects in children [5]. Evidence that PFAS exposure impairs thyroid hormone function is growing, with observed heterogeneity in associations by chemical (e.g., long-chain versus short-chain PFAS), age at exposure, and sex. Multiple epidemiologic studies of populations at both high- and low-level exposure have observed sex-specific associations between perfluorooctanoic acid (PFOA) and perfluorooctane sulfonic acid (PFOS) exposure and hypo- and hyperthyroidism [20–23]. Conversely, other studies have yielded suggestive but inconsistent evidence of alterations in thyroid hormone concentration or function [1, 24]. The hypothalamic-pituitary-thyroid axis maintains normal, circulating levels of thyroid hormones, which are critical for metabolism, temperature regulation, cognitive development, and other functions [25]. The release of thyroid stimulating hormone (TSH) initiates the synthesis and release of thyroxine (T_4_), which is converted into a more active form, triiodothyronine (T_3_). T_3_ and T_4_ bind to proteins for transport into cells, and inversely regulate TSH through a negative feedback loop. Dysregulation can reduce thyroid hormone circulation, potentially causing abnormal proliferation in the thyroid, leading to thyroid hyperstimulation, hyperplasia, and tumorigenesis [26, 27]. In vivo and in vitro toxicology studies have found PFAS can competitively bind to thyroid transport proteins and upregulate clearance enzymes, leading to both promoting and antagonist effects on thyroid hormone signaling and transport [28–31]. Perfluorohexane sulfonic acid (PFHxS) exposure in rats induced hypertrophy or hyperplasia of thyroid follicular cells [32], and PFOS and PFOA lowered total and free T_4_ concentrations [33, 34]. PFAS also have been hypothesized to increase T_4_ metabolism in the liver or thyroid, reduce thyroid peroxidase activity, and decrease T_3_ and T_4_ concentrations, which could lead to an increase in TSH and thyroid proliferation [24, 35].

Given in utero exposures may play an important role in health outcomes later in life, analysis of biological specimens collected at birth provides a valuable opportunity to quantify exposures at a critical window of development [36, 37]. Use of preclinical biospecimens also ensures the proper temporal relationship between assessments of exposures and disease, avoiding reverse causality. Neonatal dried blood spots (DBS) are collected on filter cards shortly after birth, usually 24–48 h postpartum, to screen for congenital issues [38]. Biomonitoring using DBS enables assessment of early life exposures and biological changes in the years preceding clinical manifestation of disease [36, 38–42]. Several studies have developed and validated high-performance liquid chromatography-mass spectrometry (HPLC-MS) methods for quantifying PFAS in DBS [43–48]. These studies have also addressed methodological concerns related to using DBS for PFAS biomonitoring, including demonstrating minimal PFAS background contamination in DBS cards [44, 45, 47, 48], adjusting for DBS variability, and comparing PFAS concentrations between DBS samples and fresh adult venous blood samples, reporting strong correlations between the sample types [45, 46, 48].

Several studies have examined associations between PFAS quantified in newborn DBS and potential early childhood neurotoxic, obesogenic, immunotoxic, and epigenetic effects [49–53]. However, to our knowledge, no studies have examined associations between PFAS concentrations and thyroid hormone levels in neonatal DBS. Using a newly developed method for quantifying 22 PFAS in newborn DBS [48], we conducted a pilot study to evaluate the relationships between PFAS concentrations, thyroid hormone levels, and newborn characteristics in neonatal DBS samples collected over a span of 33 years (1985–2018) for the purposes of informing future research. Additionally, to our knowledge 6 of the PFAS we examined using this method—perfluorobutanoic acid (PFBA), perfluorononane sulfonic acid (PFNS), perfluoropentane sulfonic acid (PFPeS), 4:2 fluorotelomer sulfonic acid (4:2 FTS), 6:2 FTS, and 8:2 FTS—have not been measured previously in neonatal DBS.

## Methods

### Sample acquisition

We obtained 18 neonatal DBS from the California Department of Public Health (CDPH) Newborn Screening Program, the maximum number of samples available to researchers for methods optimization studies. Demographic data accompanying samples included sex, birth year, and race/ethnicity. By request, samples were from 9 male and 9 female infants, and from different decades of birth from 1985–2018. As part of routine testing for congenital conditions at the CDPH, 5 14-mm diameter DBS are collected from newborns on filter paper by heel-stick soon after birth, typically 24–48 h after birth, and optimally by 4 days of age [54]. Typically, 2–3 DBS remain after routine screening, which have been archived by the California Newborn Screening Program since 1982. The program includes nearly all live births in California. Prior to testing, all parents were provided with a privacy notification which describes the possible research use of infant specimens, and had the opportunity to request that their newborn’s specimen not be used for such purposes [55].

### Thyroid hormone measurement and parameterization

Because hypothyroidism in neonates can lead to severe cognitive impairments in children, US newborns have been screened for congenital hypothyroidism using newborn DBS samples since the 1970s [56, 57]. We obtained thyroid hormone data from the California Newborn Screening Program for the same samples used for PFAS measurement. In California, the neonatal DBS samples were tested for T_4_ through 1997. Starting in 1998, T_4_ screening was replaced by TSH measurements because T_4_ yielded higher rates of false positives (particularly in low birthweight and preterm infants), and TSH was determined to be more specific [57, 58]. The two approaches to screening also detect different etiologies of congenital hypothyroidism: T_4_ screening better detects central hypothalamic-pituitary hypothyroidism, while TSH screening better detects subclinical hypothyroidism [59]. Because of these changes to the screening test, 8 of our pilot study samples have T_4_ measurements and 10 have TSH measurements.

To maximize use of our samples, we used three approaches to examine thyroid hormone measurements in relation to PFAS concentrations and neonatal characteristics. First, we carried out statistical analyses for T_4_ and TSH separately using concentrations measured at the time of the newborn screening test. Second, we dichotomously categorized T_4_ and TSH concentrations as normal or abnormal based on newborn screening guidelines for congenital hypothyroidism. Low T_4_ concentrations <129 nmol/l or <10 μg/dl, and high TSH concentrations >10 mU/l are considered abnormal values, triggering further testing to rule out congenital hypothyroidism [54]. Third, we converted each thyroid hormone measurement into a *z*-score so that T_4_ and TSH concentrations could be examined together and on a continuous scale. *Z*-scores were calculated by taking the difference between the observed thyroid hormone concentration and the T_4_ or TSH sample mean, and then dividing by the T_4_ or TSH sample standard deviation.

### Quantification of PFAS concentrations in dried blood spots

#### Sample preparation

DBS were sectioned into quarters using methanol-cleaned stainless-steel scissors. A quarter of each specimen was selected, measured for area and mass, and placed in a 15 ml tube, which was spiked with 10 μl of a 10 mg/ml mixture of internal standards containing 13 ^13^C_4_-labeled PFAS analytes (Wellington Laboratories Inc.) and air-dried for 30 min at room temperature. Blanks used to monitor for contamination were cut from adjacent to each DBS, area and mass measured, and processed using the same method as for DBS samples. Blood and blank samples were extracted with 1 ml methanol containing NaOH (20 mM) by shaking with 4 stainless steel beads for 20 min (1600 MiniG™ SPEX homogenizer, 1500 shakes per minute). Samples were sonicated for 10 min and centrifuged for 20 min (4000 RPM), and 500 μl of supernatant pipetted into a 2 ml polypropylene vial. The sample extraction process was repeated 3 times, with an additional 500 μl methanol with 20 mM NaOH added each time, for a total of 1500 μl supernatant. The supernatant was vortexed and dried down under nitrogen flow at 50 °C (Biotage TurboVap LV). Samples were reconstituted with 50 µl of methanol and 50 µl Mili-Q water and vortexed. Sorbent and other debris were removed by passing samples through a microcentrifuge tube cellular acetate membrane filter (Fisher Scientific) by centrifuging for 10 min (14,000 RPM). The filtrate was transferred to a polypropylene insert in a liquid chromatography mass spectrometry vial for analysis. One extraction blank and one solvent blank spiked with standards (1 ng/ml) were also extracted alongside the DBS samples to account for potential contamination in the extraction process.

#### Sample analysis

Samples were analyzed on an Ultimate 3000 ultra-high-performance liquid chromatograph system, coupled to a Q-Exactive high-resolution orbitrap mass spectrometer (Thermo Scientific) [60–62]. The mobile phase was composed of A (0.1% formic acid in ultra-pure water) and B (0.1% formic acid in acetonitrile). A Restek PFAS delay column (50 mm × 2.1 mm, 5 um particles) and a Thermo Hypersil Gold C-18 column (100 mm × 2.1 mm, 1.9 µm particles) with an Accucore Q guard column (10 mm × 2.1 mm, 2.6 μm particles) were used for chromatographic separation using gradient separations of 20% B (0–2.5 min) and then 30–85% B (2.5–19 min). Between runs, there was a gradient column rinse (20% B at 19 min, up to 100% B at 20.5 min) and re-equilibration with 20% B (20.5–23 min). The injection volume was 10 μl; flow rate 300 μl/min; column oven maintained at 40 °C; and autosampler maintained at 10 °C. Quality control and instrument blank samples were run every 8–12 samples. A six-point calibration curve (0.01, 0.05, 0.1, 0.5, 1, 2 ng/ml) with internal standards (1 ng/ml) in a 50:50 methanol:water solution was run alongside samples. Additional methods parameters have been published previously [48].

#### PFAS quantification

We analyzed each DBS for 22 PFAS, provided in Supplementary Table S1. Nine PFAS had at least one sample with a concentration >LOD. Of these, 6:2 fluorotelomer sulfonic acid (6:2 FTS), was excluded from further analysis because of challenges with recovery. The 8 remaining PFAS measured were PFOA, PFOS, perfluorooctane sulfonamide (PFOSA), PFBA, PFBS, perfluoroheptanoic acid (PFHpA), perfluoroheptane sulfonic acid (PFHpS), and PFHxS. Full MS scans with exact mass (Δ *m*/*z* ≤ 10 ppm) were used for PFAS identification and quantification. Calibration was based on an isotope dilution strategy and curves were weighted 1/x. Limits of detection for this method have previously been determined [48] and are as follows: PFOA, 0.083 ng/ml; PFOS, 0.090 ng/ml; PFOSA, 0.014 ng/ml; PFBA, 0.25 ng/ml; PFBS, 0.027 ng/ml; PFHpA, 0.060 ng/ml; PFHpS, 0.0080 ng/ml; PFHxS, 0.011 ng/ml.

#### PFAS concentration normalization

To account for both (1) heterogeneity in blood spot area and volume and (2) potential contamination of the collection cards with PFAS [44, 63, 64], we applied a multi-step normalization approach using paired PFAS measurements in DBS samples and card blank material as previously described [48]. Briefly, PFAS concentrations (ng/ml) were quantified in the extracts from paired DBS and card blanks. The card blank sample collected was adjacent to and of equal area to the DBS sample. We assumed uniform density of the card material and calculated the mass of the paper and blood in each DBS sample based on the mass of the card blanks. PFAS detected in card blanks were assumed to be present in the DBS at the same level on a mass per card area basis, and levels in card blanks were subtracted from DBS measurements. DBS measurements above the LOD were included in further analyses if the PFAS was not detected in the paired card blank or if the DBS measurement was at least 20% higher than the paired card blank (a criterion informed by previous repeatability testing); all samples satisfied this criterion (Supplementary Table S2). Final PFAS concentrations are reported in units of pg PFAS/g dried blood. An example calculation is available in the Supplementary Material. Concentrations were reported on the basis of dried blood mass to account for variability in the amount of blood absorbed by different types of collection cards.

#### PFAS exposure assignment

In addition to continuous PFAS concentrations, we created binary PFAS variables for use in statistical comparisons with thyroid hormone concentrations and neonatal characteristics. We used the median number of detected PFAS (*n* = 3) to create a binary categorical variable describing samples with either a low number of PFAS detected (0–2) or a higher number of PFAS detected (≥3).

### Statistical analysis

We described the distribution of newborn characteristics, including year of birth by decade, number of hours after birth when DBS collection occurred (in three groups of ≤24, 25–48, and 49–72 h), infant sex, and infant race/ethnicity. We calculated median and interquartile ranges (IQR) of T_4_ (nmol/l), TSH (mU/l), and PFAS (pg/g) concentrations across all subjects and stratified by newborn characteristics, with non-parametric two-sided Wilcoxon rank-sum and Kruskal–Wallis tests used to compare differences across decade of birth and DBS collection time. Spearman correlation coefficients were used to assess the relationships between those PFAS with at least 50% of samples above the LOD (PFOA, PFOS, and PFOSA), and between thyroid hormone levels and PFOA, PFOS, and PFOSA. We also examined participant characteristics (decade of birth, sex, race/ethnicity, thyroid hormone concentrations, and thyroid hormone *z*-scores) stratified by the categorical PFAS exposure variables. Because of small group sizes, we consider stratified analyses by infant sex and race/ethnicity to be secondary analyses and have not carried out formal statistical comparisons across the groups. All analyses were carried out in SAS, version 9.4. We used an alpha value of 0.05 for statistical significance; however, due to the pilot nature of this study and small sample size, we do not strictly interpret results using null hypothesis significance testing, but rather attempt to determine if results are compatible with relationships between various factors and are biologically plausible [65].

## Results

### Participant characteristics

The study population included 9 female and 9 male infants (Table 1). A total of 6 infants were identified as White (33%), 3 were Hispanic (17%), 3 were Asian (17%), 2 were Black (11%), and 4 were of unknown race/ethnicity (22%). The birth years of the 18 infants ranged from 1985 to 2018. Most DBS sampling (13 samples or 72%) was carried out 24 to 72 h after birth, and within 24 h after birth for 5 (28%) infants.

**Table 1:** Thyroid hormone concentrations in 18 neonatal dried blood spots stratified by study participant characteristics.

Participant characteristics	TSH (mU/l), median (IQR), *n*	T_4_ (nmol/l), median (IQR), *n*	Normal NBS, *n*^a^	Abnormal NBS, *n*^a^	Thyroid hormone *z*-score, median (IQR)^b^
Full cohort	5.5 (2.7, 6.3), *n* = 10	138 (104, 147), *n* = 8	15	3	0.09 (−0.98, 0.41)
Decade of birth					
1985–1994 (*n* = 5)	–	139 (92, 142)	3	2	0.07 (−1.02, 0.14)
1995–2004 (*n* = 7)	3.9 (1.7, 5.6), *n* = 4	137 (116, 222), *n* = 3	6	1	0.02 (−0.98, 0.41)
2005–2014 (*n* = 2)	4.1 (2.4, 5.7)	–	2	0	−0.39 (−1.07, 0.29)
≥ 2015 (*n* = 4)	6.8 (5.8, 7.9)	–	4	0	0.73 (0.33, 1.21)
	*p* = 0.10^c^	*p* = 0.79^d^			*p* = 0.20^c^
DBS collection time (hours after birth)					
≤24 h (*n* = 5)	6.0 (5.5, 7.5), *n* = 4	222, *n* = 1	5	0	0.55 (0.27, 1.50)
>24–48 h (*n* = 7)	5.6 (3.9, 6.6), *n* = 4	137 (116, 152), *n* = 3	6	1	0.11 (−0.48, 0.41)
>48–72 h (*n* = 6)	1.7 (0.7, 2.7), *n* = 2	115.5 (90, 140.5), *n* = 4	4	2	−1.01 (−1.14, 0.07)
	*p* = 0.16^c^	*p* = 0.26^c^			*p* = 0.02^c^
Infant sex					
Male (*n* = 9)	5.3 (2.7, 6.0), *n* = 5	104 (90, 127.5), *n* = 4	6	3	−0.48 (−1.05, 0.11)
Female (*n* = 9)	5.7 (5.2, 6.3), *n* = 5	147 (139.5, 187), *n* = 4	9	0	0.29 (0.08, 0.55)
	*p* = 0.84^d^	*p* = 0.06^d^			*p* = 0.14^d^
Race/ethnicity					
White (*n* = 6)	5.7 (5.2, 6.0); *n* = 5	152; *n* = 1	6	0	0.33 (0.08, 0.41)
Hispanic (*n* = 3), Black (*n* = 2), Asian (*n* = 3)	5.3 (2.4, 7.2); *n* = 5	137 (116, 222); *n* = 3	7	1	0.07 (−0.77, 1.21)
Unknown (*n* = 4)	–	115.5 (90.0, 140.5); *n* = 4	2	2	−0.49 (−1.10, 0.11)
	*p* = 1.00^d^	*p* = 0.40^c^			*p* = 0.33^c^

*TSH* thyroid stimulating hormone, *T*_*4*_ thyroxine, *IQR* interquartile range, *NBS* newborn screening test, *DBS* dried blood spot, *Q1–Q4* first through fourth quartiles.

^a^TSH and T_4_ concentrations were classified as normal or abnormal based on newborn screening test guidelines for congenital hypothyroidism. Abnormal T_4_ concentrations are <129 nmol/l or <10 μg/dl, and abnormal TSH concentrations are >10 mU/l.

^b^Thyroid hormone *z*-scores were calculated by taking the difference between the observed thyroid hormone concentration and the T_4_ or TSH sample mean, and then dividing by the T_4_ or TSH sample standard deviation. The T_4_ sample mean was 136.0 nmol/l and sample standard deviation was 42.0 nmol/l; the TSH sample mean was 5.0 mU/l and sample standard deviation was 2.4 mU/l.

^c^*p* values are from a two-sided Kruskal–Wallis test comparing thyroid hormone concentrations or *z*-scores across the participant characteristic groups.

^d^*p* values are from a two-sided Wilcoxon rank-sum test comparing thyroid hormone concentrations or *z*-scores across the participant characteristic groups.

### Thyroid hormone concentrations

**Fig. 1:**
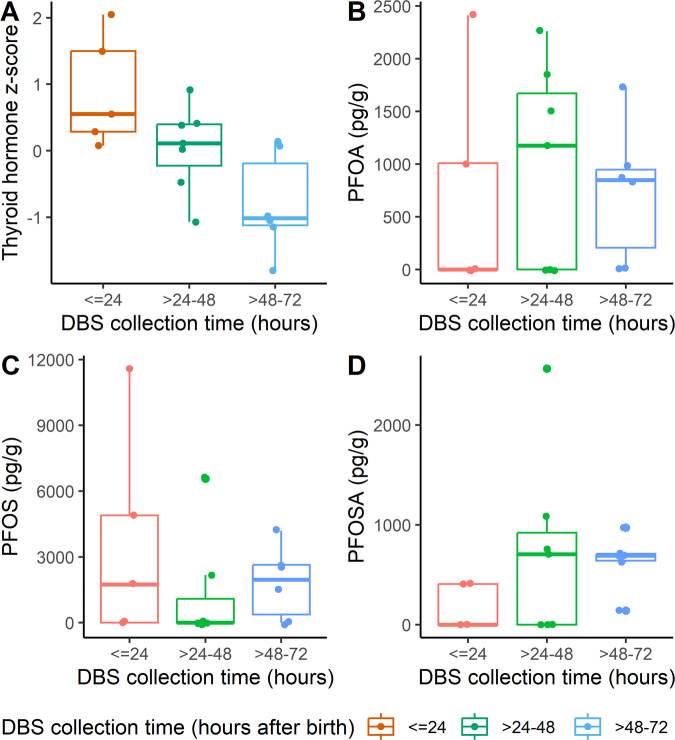
Thyroid hormone *z*-scores and PFAS concentrations (pg/g) by DBS collection time (hours after birth). **A** Thyroid hormone z-scores stratified by DBS collection time. **B** PFOA concentrations (pg/g) stratified by DBS collection time. **C** PFOS concentrations (pg/g) stratified by DBS collection time. **D** PFOSA concentrations (pg/g) stratified by DBS collection time. Boxplots show the median, 25th percentile, and 75th percentile values, with whiskers extending to the minimum and maximum values. Only those PFAS with at least 50% of sample concentrations >LOD are included, with concentrations <LOD treated as LOD/2. The limits of detection for the PFAS are: LOD_PFOA_ = 0.083 ng/ml, LOD_PFOS_ = 0.090 ng/ml, and LOD_PFOSA_ = 0.014 ng/ml. DBS dried blood spot, PFAS per- and polyfluoroalkyl substances, PFOA perfluorooctanoic acid, PFOS perfluorooctane sulfonic acid, PFOSA perfluorooctane sulfonamide, LOD limit of detection.

TSH concentrations for the most recent samples were higher compared to previous decades, but the T_4_ concentrations were similar in the decades assessed (Table 1). TSH and T_4_ concentrations declined as the DBS collection time (hours after birth) increased; this decreasing trend was clear and statistically significant when analyzed as *z*-scores (Table 1 and Fig. 1). While TSH concentrations were similar for male and female infants, T_4_ concentrations were higher for females. According to newborn screening guidelines, there were three abnormal T_4_ concentrations, all from males, and no abnormal TSH concentrations. Thyroid hormone concentrations were similar across different race/ethnicity groups.

### PFAS concentrations

PFAS concentrations were above the LOD for more than 50% of samples for PFOA, PFOS, and PFOSA (Table 2). For PFBA, PFBS, PFHpA, PFHpS, and PFHxS, less than 50% of samples had concentrations above the LOD. Correlations between concentrations of PFOA, PFOS, and PFOSA ranged from 0.17 to 0.40, with the strongest correlation between PFOA and PFOS (Spearman’s *r* = 0.40) (Table 3). Concentrations of PFOA, PFOS, and PFOSA were lower in more recently collected samples, while PFBA was elevated in the most recent decade (Table 2). However, only PFOSA concentrations exhibited a statistically significant difference across the decades evaluated by this study (*p* = 0.006). Due to low detection frequencies and small sample size, it was not possible to assess potential trends in concentration over time for the other PFAS. The frequency of detection generally declined for PFOA, PFOS, PFOSA, and PFBS in more recent decades, increased for PFBA, and fluctuated for PFHpA and PFHxS, approximately mirroring potential temporal trends in concentration. There was no statistically significant difference in PFAS concentration with respect to sample collection time (Fig. 1), and concentrations did not appear to differ by sex or race/ethnicity (Table S3). Examining participant characteristics stratified by the PFAS exposure metric, more recent samples appeared to have a lower number of detectable PFAS compared to samples from earlier decades (Tables 4 and S4).

**Table 2:** PFAS concentrations (pg/g) in 18 neonatal dried blood spots stratified by study participant characteristics.

Participant or sample characteristic	PFOA, median (IQR)	PFOS, median (IQR)	PFOSA, median (IQR)	PFBA, median (IQR)	PFBS, median (IQR)	PFHpA, median (IQR)	PFHpS, median (IQR)	PFHxS, median (IQR)
Full cohort (*n* = 18)	848.2 (<LOD, 1503.7)	739.1 (<LOD, 2705.5)	519.5 (<LOD, 715.3)	<LOD	<LOD (<LOD, 941.6)	<LOD	<LOD	<LOD (<LOD, 184.6)
Samples >LOD, *n* (%)	10 (56%)	9 (50%)	12 (67%)	4 (22%)	8 (44%)	1 (6%)	4 (22%)	5 (28%)
LOD (ng/ml)	0.083	0.090	0.014	0.25	0.027	0.060	0.0080	0.011
Half-life^a^	1.8–3.8 years	2.9–4.8 years	1.7 years	3 days	26–44 days	62–70 days	1.5 years	2.9–5.3 years
Decade of birth								
1985–1994 (*n* = 5)	872.8 (823.6, 1736.9)	2446.0 (<LOD, 2705.5)	715.3 (687.3, 971.3)	<LOD	1095.4 (<LOD, 4091.2)	<LOD	<LOD	<LOD (<LOD, 241.5)
1995–2004 (*n* = 7)	972.1 (<LOD, 1839.8)	1478.2 (<LOD, 4891.3)	694.4 (407.7, 758.4)	<LOD	<LOD (<LOD, 941.6)	<LOD	<LOD (<LOD, 127.4)	<LOD (<LOD, 509.0)
2005–2014 (*n* = 2)	751.8 (<LOD, 1503.7)	1955.7 (1741.4, 2170.1)	<LOD	<LOD	274.5 (<LOD, 549.0)	597.8 (<LOD, 1195.7)	<LOD	<LOD
≥2015 (*n* = 4)	<LOD (<LOD, 588.3)	<LOD	<LOD	1381.2 (<LOD, 3175.4)	<LOD (<LOD, 253.1)	<LOD	<LOD	<LOD
	*p* = 0.60^b^	*p* = 0.24^b^	*p* = 0.0064^b^	*p* = 0.46^b^	*p* = 0.46^b^	*p* = 0.046^b, c^	*p* = 0.42^b^	*p* = 0.37^b^
DBS collection time (hours after birth)								
≤24 h (*n* = 5)	<LOD (<LOD, 1000.8)	1741.4 (<LOD, 4891.3)	<LOD (<LOD, 407.7)	<LOD (<LOD, 2636.2)	<LOD	<LOD	<LOD (<LOD, 127.4)	<LOD
>24–48 h (*n* = 7)	1176.6 (<LOD, 1839.8)	<LOD (<LOD, 2170.1)	704.6 (<LOD, 1083.3)	<LOD	549.0 (<LOD, 1536.5)	<LOD	<LOD	<LOD
>48–72 h (*n* = 6)	848.2 (<LOD, 972.1)	1962.1 (<LOD, 2705.5)	690.9 (625.2, 715.3)	<LOD	<LOD (<LOD, 1095.4)	<LOD	<LOD	92.3 (<LOD, 241.5)
	*p* = 0.80^b^	*p* = 0.47^b^	*p* = 0.12^b^	*p* = 0.71^b^	*p* = 0.16^b^	*p* = 0.46^b^	*p* = 0.55^b^	*p* = 0.56^b^

*PFAS* per- and polyfluoroalkyl substances, *PFOA* perfluorooctanoic acid, *PFOS* perfluorooctane sulfonic acid, *PFOSA* perfluorooctane sulfonamide, *PFBA* perfluorobutanoic acid, *PFBS* perfluorobutane sulfonic acid, *PFHpA* perfluoroheptanoic acid, *PFHpS* perfluoroheptane sulfonic acid, *PFHxS* perfluorohexane sulfonic acid, *IQR* interquartile range, *LOD* limit of detection, *DBS* dried blood spot, *Q1–Q4* first through fourth quartiles, *TSH* thyroid stimulating hormone, *T*_*4*_ thyroxine.

^a^PFAS serum half-lives in humans as reported in Chang et al. [66], Olsen et al. [8], Spliethoff et al. [45], Xu et al. [9].

^b^*p* values are from a two-sided Kruskal–Wallis test comparing thyroid hormone concentrations or *z*-scores across the participant characteristic groups, with concentrations <LOD treated as LOD/2.

^c^Only one sample of PFHpA was >LOD, so this *p* value should not be considered indicative of a statistically significant trend.

**Table 3:** Spearman correlations between PFAS concentrations (pg/g) and thyroid hormone concentrations in 18 neonatal dried blood spots.

	PFOA	PFOS	PFOSA
PFOA	1	0.40	0.20
PFOS		1	0.17
PFOSA			1
T_4_	0.59	0.49	0.19
TSH	−0.40	−0.70	−0.36

PFAS concentrations <LOD were substituted using LOD/2. Only those PFAS with at least 50% of samples >LOD are included in this analysis.

*PFAS* per- and polyfluoroalkyl substances, *PFOA* perfluorooctanoic acid, *PFOS* perfluorooctane sulfonic acid, *PFOSA* perfluorooctane sulfonamide, *TSH* thyroid stimulating hormone, *T*_*4*_ thyroxine, *LOD* limit of detection.

**Table 4:** Participant characteristics of 18 infants stratified by the number of different PFAS detected in individual dried blood spot samples.

Participant characteristics	Participants with 0–2 PFAS detected, *n*^a^	Participants with 3–6 PFAS detected, *n*^a^	*p* value^b^
Full cohort	8	10	–
Decade of birth			
1985–1994	1	4	–
1995–2004	2	5	–
2005–2014	1	1	–
≥2015	4	0	–
Thyroid hormone *z*-score, median (IQR)	0.35 (−0.18, 0.73)	0.05 (−1.07, 0.14)	0.12
TSH (mU/l), median (IQR) (*n* = 10)	6.17 (5.70, 7.21)	2.55 (1.56, 3.93)	0.01
T_4_ (nmol/l), median (IQR) (*n* = 8)	104 (92, 116)	140.5 (137.0, 152.0)	0.29
Newborn screening test results^c^			
Normal thyroid hormone level	6	9	–
Abnormal thyroid hormone level	2	1	–

*PFAS* per- and polyfluoroalkyl substances, *IQR* interquartile range, *TSH* thyroid stimulating hormone, *T*_*4*_ thyroxine.

^a^The median number of PFAS detected in cohort samples was 3.

^b^*p* values are from a two-sided Wilcoxon rank-sum test comparing thyroid hormone concentrations across the PFAS exposure metric categories.

^c^TSH and T_4_ concentrations were classified as normal or abnormal based on newborn screening test guidelines for congenital hypothyroidism. Abnormal T_4_ concentrations are <129 nmol/l or <10 μg/dl, and abnormal TSH concentrations are >10 mU/l.

### Relationships between PFAS and thyroid hormone concentrations

Spearman correlations between PFOA, PFOS, and PFOSA and thyroid hormone concentrations ranged from −0.70 to 0.59 (Table 3). Correlations between PFAS and T_4_ were positive, while correlations between PFAS and TSH were negative. PFOA was most strongly correlated with T_4_ (*r* = 0.59), and PFOS was most strongly correlated with TSH (*r* = −0.70). Concentrations of PFAS were stratified by different classifications of thyroid hormone measurements based on newborn screening guidelines in Table 5, but low detection frequencies make it difficult to observe clear trends. No differences were observed in PFAS concentrations between newborns with normal or abnormal thyroid hormone levels overall, in relation to normal versus abnormal TSH (all samples had normal TSH concentrations), or for thyroid hormone *z*-scores. However, when T_4_ was classified as normal or abnormal based on newborn screening guidelines, median concentrations of PFOA, PFOS, and PFBS appeared lower in samples with abnormal/low T_4_ levels. In addition, samples in which 3–6 different PFAS were detected had lower TSH (*p* = 0.01) compared to samples in which 0–2 PFAS were detected (Table 4). T_4_ concentrations (*p* = 0.29) and *z*-scores (*p* = 0.12) were not statistically significantly different for samples with 0–2 PFAS compared to those with 3–6 PFAS.

**Table 5:** PFAS concentrations (pg/g) stratified by thyroid hormone concentrations in 18 neonatal dried blood spots^a^.

Thyroid hormone measurement	PFOA, median (IQR)	PFOS, median (IQR)	PFOSA, median (IQR)	PFBA, median (IQR)	PFBS, median (IQR)	PFHpA, median (IQR)	PFHpS, median (IQR)	PFHxS, median (IQR)
Newborn screening test results								
Normal thyroid hormone level (*n* = 15)	972.1 (<LOD, 1736.9)	1478.2 (<LOD, 4189.1)	407.7 (<LOD, 715.3)	<LOD (<LOD, <LOD)	<LOD (<LOD, 941.6)	<LOD (<LOD, <LOD)	<LOD (<LOD, 32.9)	<LOD (<LOD, 184.6)
Abnormal thyroid hormone level (*n* = 3)	<LOD (<LOD, 872.8)	<LOD (<LOD, 2705.5)	687.3 (625.2, 758.4)	<LOD (<LOD, 4057.6)	<LOD (<LOD, 1095.4)	<LOD	<LOD	<LOD (<LOD, 241.5)
Thyroid hormone concentrations^b^								
T_4_ Normal (*n* = 5)	1736.9 (1008.8, 1839.8)	2446.0 (<LOD, 6569.6)	715.3 (704.6, 971.3)	<LOD	941.6 (378.2, 4091.2)	<LOD	<LOD (<LOD, 173.7)	<LOD
T_4_ Abnormal, <129 nmol/l (*n* = 3)	<LOD (<LOD, 872.8)	<LOD (<LOD, 2705.5)	687.3 (625.2, 758.4)	<LOD (<LOD, 4057.6)	<LOD (<LOD, 1095.4)	<LOD	<LOD	<LOD (<LOD, 241.5)
TSH Normal (*n* = 10)	<LOD (<LOD, 1176.6)	739.1 (<LOD, 2170.1)	<LOD (<LOD, 413.9)	<LOD (<LOD, 2636.2)	<LOD (<LOD, 506.3)	<LOD	<LOD	<LOD (<LOD, 184.6)
TSH Abnormal, >10 mU/l (*n* = 0)	–	–	–	–	–	–	–	–
Thyroid hormone *z*-score								
Q1, *z* ≤ −0.97 (*n* = 4)	922.5 (436.4, 1237.9)	1824.2 (739.1, 2437.8)	383.2 (70.6, 656.3)	<LOD (<LOD, 2028.8)	274.5 (<LOD, 822.2)	<LOD (<LOD, 597.8)	<LOD (<LOD, 16.4)	92.3 (<LOD, 213.1)
Q2–3, −0.98 ≤ *z* < 0.41 (*n* = 9)	823.6 (<LOD, 1839.8)	1741.4 (<LOD, 4189.1)	704.6 (413.9, 758.4)	<LOD	<LOD (<LOD, 941.6)	<LOD	<LOD	<LOD (<LOD, 509.0)
Q4, *z* ≥ 0.41 (*n* = 5)	<LOD (<LOD, 1008.8)	<LOD	<LOD (<LOD, 407.7)	<LOD	<LOD (<LOD, 378.2)	<LOD	<LOD	<LOD

*PFAS* per- and polyfluoroalkyl substances, *PFOA* perfluorooctanoic acid, *PFOS* perfluorooctane sulfonic acid, *PFOSA* perfluorooctane sulfonamide, *PFBA* perfluorobutanoic acid, *PFBS* perfluorobutane sulfonic acid, *PFHpA* perfluoroheptanoic acid; *PFHpS* perfluoroheptane sulfonic acid, *PFHxS* perfluorohexane sulfonic acid, *IQR* interquartile range, *LOD* limit of detection, *DBS* dried blood spot, *Q1–Q4* first through fourth quartiles, *TSH* thyroid stimulating hormone, *T*_*4*_ thyroxine.

^a^The limits of detection for the PFAS are: LOD_PFOA_ = 0.083 ng/ml, LOD_PFOS_ = 0.090 ng/ml, LOD_PFOSA_ = 0.014 ng/ml, LOD_PFBA_ = 0.25 ng/ml, LOD_PFBS_ = 0.027 ng/ml, LOD_PFHPA_ = 0.060 ng/ml, LOD_PFHPS_ = 0.0080 ng/ml, and LOD_PFHxS_ = 0.011 ng/ml.

^b^TSH and T_4_ concentrations were classified as normal or abnormal based on newborn screening test guidelines for congenital hypothyroidism. Abnormal T_4_ concentrations are <129 nmol/l or <10 μg/dl, and abnormal TSH concentrations are >10 mU/l.

## Discussion

In this pilot study, we examined the relationship between PFAS concentrations, thyroid hormone levels, and neonatal characteristics in newborn DBS collected over three decades. While PFAS have previously been quantified in DBS, and thyroid hormones are routinely measured in newborn DBS to screen for congenital hypothyroidism, to our knowledge, this is the first study to examine both PFAS and thyroid hormone concentrations using DBS, and their relationships with neonatal characteristics. To our knowledge, it is also the first study to attempt to measure PFBA, PFNS, PFPeS, 4:2 FTS, 6:2 FTS, and 8:2 FTS in newborn DBS. We observed that the concentration and detection frequency of PFOA, PFOS, and PFOSA were generally lower in more recently collected samples compared to the earliest years assessed by this study. The time of specimen collection after birth was related to thyroid hormone concentrations. PFAS concentrations were unrelated to time of sample collection, indicating that PFAS are not sensitive to immediate postpartum physiologic changes or post-birth exposures to PFAS occurring in the hours before DBS sampling. Among PFAS with at least 50% of samples >LOD, we found that thyroid hormone measurements were correlated with concentrations of PFOA and PFOS. Our results provide valuable information for biomonitoring studies using neonatal DBS and suggest directions for future research on potential relationships between PFAS exposure and thyroid hormones.

The decline in TSH and T_4_ concentrations as the DBS sample collection time increased is consistent with normal physiologic changes occurring soon after birth. At birth, exposure of the newborn’s skin to a cooler environment and cutting the umbilical cord stimulate a catecholamine surge, with TSH peaking 15–60 min postpartum [59]. TSH levels decline to 50% of the peak by 2 h postpartum, 20% of the peak by 24 h postpartum, and continue to decline over the next 2–3 days. The post-birth TSH surge stimulates an increase in T_4_, which peaks at 24–36 h postpartum and declines over the next few weeks [59]. For these reasons, most screening occurs between 24 and 48 h after birth and by 4 days of age, timed to ascertain thyroid hormone levels after post-birth surges have begun to normalize in order to minimize false negatives and positives in congenital hypothyroidism screening [54]. However, we observed that time of sampling is still strongly correlated with both TSH and T_4_ levels, even within the recommended timeframe (Table 1 and Fig. 1). This finding underscores the importance of accounting for the time of sample collection in studies using thyroid hormone concentrations from newborn DBS.

In contrast to the patterns observed with thyroid hormone levels, the time of sample collection was unrelated to PFAS concentrations (Table 2 and Fig. 1), suggesting that PFAS concentrations are not sensitive to physiological changes during the immediate postpartum period and providing support for the use of PFAS concentrations measured in newborn DBS as an indicator of in utero exposures. This is consistent with reported serum half-lives in humans of the PFAS measured in this study, which range from 1.5 to 5 years for PFOA, PFOS, PFOSA, PFHpS, and PFHxS, and 3 to 70 days for PFBA, PFBS, and PFHpA (Table 2) [8, 9, 45, 66]. Together with longer half-lives, the stability of PFAS in relation to DBS sample timing supports the validity of using DBS to estimate neonatal PFAS exposures occurring during pregnancy.

The median concentrations of PFOS and PFOA reported in this study are similar to those reported in the limited number of existing studies that have evaluated newborn DBS, particularly when considering samples collected during similar years [43–45, 53]. Serum concentrations of PFAS have changed over the past decades, with the direction of temporal trends varying by chemical. In adult and child populations worldwide, serum concentrations of PFOS and PFOA increased from the 1970s to the 1990s, and began decreasing in 2000 due to regulatory restrictions and voluntary phase-outs [5, 67–69]. Our observation of lower concentrations of PFOA, PFOS, and PFOSA in more recent decades is consistent with these global trends. Similar findings were also reported by a study conducted in New York State using 2640 neonatal DBS from 1997 to 2007 [45]. Maximum concentrations of PFOS, PFOSA, PFOA, and PFHxS occurred between 1998 and 2001, and steadily declined after 2001 [45]. We also found fewer PFAS (of the 8 compounds assessed) were detected in more recent samples (Tables 2 and 4). The exception was PFBA, which was only detectable in the most recent time period, from 2015 to 2018. Exposures to other PFAS, such as PFHxS and perfluorononanoic acid (PFNA), have also increased through the 2000s [67, 70, 71]. The lack of regulation of PFAS as a chemical class has resulted in temporal variation that differs compound by compound. Individual legacy PFAS subject to regulation have declined while alternative and replacement PFAS have increased in use and exposures over time [5]. Because of our pilot study’s limited sample size, the temporal trends in PFAS concentrations we observed—and their concordance with trends in larger studies—primarily serve to support the potential representativeness of our samples, rather than to precisely quantify the trends. The correlations between PFOA and PFOS in DBS that we observed are similar to those reported in National Health and Nutrition Examination Survey (NHANES) samples from a similar time period [72]. Data from NHANES 2003–2004 also found a correlation between PFOS and PFOA (Pearson’s *r* = 0.66) [72], which may indicate similarities in their commercial use.

To our knowledge, this pilot study is the first study to use newborn DBS to examine PFAS exposure and thyroid hormone concentrations. Several previous studies have examined associations between PFAS concentrations in maternal serum collected during pregnancy or cord blood, and thyroid hormone levels in neonatal DBS in cohorts from Norway, the Netherlands, Belgium, and the United States [73–77]. Studies from two cohorts used newborn DBS for T_4_ measurement and first trimester maternal serum and cord blood for PFAS exposure assessment [73, 75, 76]. These studies observed higher PFOA, PFOS, PFNA, and PFHxS concentrations and lower T_4_, but this relationship rarely reached statistical significance [73, 75, 76]. These associations were often sex-specific, with stronger associations for male infants. Of the two studies examining TSH from newborn DBS in relation to PFAS, one found a consistent inverse association that did not reach statistical significance, with higher cord blood concentrations of PFNA, PFOA, and PFOS and decreased TSH [77], and the other found no associations between second trimester maternal serum PFAS concentrations and TSH concentrations [74].

Research on the directionality of correlations between PFAS concentrations and thyroid hormone levels has yielded varying results [1]. In our study, concentrations of PFOA, PFOS, and PFBS appeared to be lower in samples with abnormal/low T_4_ levels (Table 5). Similarly, we found that correlations between PFAS and T_4_ concentrations were positive while correlations between PFAS and TSH were negative (Table 3). The opposing directions of correlation coefficients between PFAS concentrations and the two thyroid hormones may reflect the function of the hypothalamic-pituitary-thyroid axis, in which TSH levels are inversely correlated with T_4_ levels. TSH stimulates secretion of thyroid hormones, including T_4_, from the thyroid gland, and these thyroid hormones then reduce TSH secretion via a negative feedback loop [78].

Sex differences in associations between PFAS concentrations and thyroid hormone levels have been reported in previous studies. De Cock et al. [76] found that male but not female infants with elevated cord blood PFOS concentrations had lower T_4_ levels; this association attenuated after adjustment, and was not present with PFOA [76]. Similarly, Preston et al. found prenatal maternal concentrations of PFOS, PFOA, and PFHxS were inversely associated with T_4_ levels in male but not female infants, including in a PFAS mixtures analysis [73, 75]. In our cohort, T_4_ levels were lower for male infants (*p* = 0.06), and the 3 abnormal thyroid screens were all in male infants and based on T_4_ levels.

The strengths of this pilot study include assessment of a racially and ethnically diverse cohort sampled from a period spanning 33 years. This is the first study to use newborn DBS to quantify PFAS that also leverages available thyroid hormone measurements from the same DBS, and the first study to consider an expanded panel of PFAS, including 6 not previously quantified in newborn DBS. Results from this study address several critical methodological issues for application in future larger studies. The limitations of this study include its small sample size. This study was conducted as a pilot study, which precluded more complex modeling of PFAS-thyroid hormone relationships. While the relationships observed have been contextualized, these findings may be subject to confounding or due to chance. Samples may not be representative of the larger population. Descriptions of temporal trends for PFAS were limited by our sample size and many samples being below the LOD; as such, these patterns should be examined in larger cohorts. Additionally, it is unknown whether the abnormal newborn screening test results in this cohort were later diagnosed as cases of congenital hypothyroidism, as we do not have access to follow-up testing results. Future studies could seek to link newborn screening test results with follow-up testing and subsequent diagnosis.

Findings of this pilot study suggest that several PFAS warrant further investigation in relation to thyroid hormone levels, notably PFOA and PFOS in relation to T_4_, and PFOS in relation to TSH. Future studies with larger sample sizes could explore the opposing directions of the associations between T_4_ and TSH and various PFAS, and mechanistic studies could investigate the implications of this for the potential mechanisms of PFAS-induced thyroid hormone disruption. Additionally, this pilot study offers several methodological lessons for future research using newborn DBS to examine PFAS and thyroid hormone concentrations. Although the filter paper used in DBS stabilizes many analytes, and PFAS are persistent compounds with longer half-lives, future studies designed to investigate the stability of PFAS and other environmental chemicals in DBS after long-term storage would be informative as researchers increasingly leverage archived DBS for environmental health research. In relation to epidemiologic analyses, future studies should account for the time of DBS sample collection, which influences thyroid hormone levels even when samples are collected during the specified timeframe for newborn screening tests. Studies using archived newborn DBS across multiple decades may also encounter the challenge of harmonizing TSH and T_4_ measurements. When using the *z*-score approach to combine both T_4_ and TSH measurements, abnormal thyroid hormone concentrations will have both low and high *z*-scores; as a result, associations with PFAS concentrations or other exposures of interest could have a U-shaped distribution, and so statistical models should be able to accommodate nonlinear associations. Despite some challenges in using archived newborn DBS in environmental health studies, these samples offer unique and powerful opportunities to interrogate preclinical, population-based samples for a range of environmental chemicals, offering great potential for children’s health research [42].

## Conclusion

This pilot study uses newborn DBS to measure a panel of 8 PFAS, demonstrating the feasibility of quantifying PFAS in archived newborn DBS. This study also leverages thyroid hormone concentrations previously measured as part of routine newborn screening to highlight several methodological considerations for future studies using thyroid hormone screening data and measurements from newborn DBS. Although exploratory, potential relationships between thyroid hormones and PFAS exposure were observed. These findings should be examined in a larger cohort with a broader range of thyroid hormone measurements to more thoroughly describe potential patterns of association.

### Supplementary information

Supplementary Material

## Data Availability

The datasets generated and analyzed during the current study are generated from the California Biobank and are the property of the State of California. We are therefore unable to share the data. Researchers who would like to use the data can contact the California Department of Public Health Institutional Review Board to seek approval to utilize the data, which can then be shared peer-to-peer.

## References

[1] Zhang L, Liang J, Gao A (2023). Contact to perfluoroalkyl substances and thyroid health effects: a meta-analysis directing on pregnancy. Chemosphere.

[2] Boone JS, Vigo C, Boone T, Byrne C, Ferrario J, Benson R (2019). Per- and polyfluoroalkyl substances in source and treated drinking waters of the United States. Sci Total Environ.

[3] Glassmeyer ST, Furlong ET, Kolpin DW, Batt AL, Benson R, Boone JS (2017). Nationwide reconnaissance of contaminants of emerging concern in source and treated drinking waters of the United States. Sci Total Environ.

[4] Domingo JL, Nadal M (2017). Per- and polyfluoroalkyl substances (PFASs) in food and human dietary intake: a review of the recent scientific literature. J Agric Food Chem.

[5] Sunderland EM, Hu XC, Dassuncao C, Tokranov AK, Wagner CC, Allen JG (2019). A review of the pathways of human exposure to poly- and perfluoroalkyl substances (PFASs) and present understanding of health effects. J Expo Sci Environ Epidemiol.

[6] Savvaides T, Koelmel JP, Zhou Y, Lin EZ, Stelben P, Aristizabal-Henao JJ (2021). Prevalence and implications of per- and polyfluoroalkyl substances (PFAS) in settled dust. Curr Environ Heal Rep.

[7] DeLuca NM, Minucci JM, Mullikin A, Slover R, Cohen Hubal EA (2022). Human exposure pathways to poly- and perfluoroalkyl substances (PFAS) from indoor media: a systematic review. Environ Int.

[8] Olsen GW, Burris JM, Ehresman DJ, Froehlich JW, Seacat AM, Butenhoff JL (2007). Half-life of serum elimination of perfluorooctanesulfonate, perfluorohexanesulfonate, and perfluorooctanoate in retired fluorochemical production workers. Environ Health Perspect.

[9] Xu Y, Fletcher T, Pineda D, Lindh CH, Nilsson C, Glynn A (2020). Serum half-lives for short-and long-chain perfluoroalkyl acids after ceasing exposure from drinking water contaminated by firefighting foam. Environ Health Perspect.

[10] Sagiv SK, Rifas-Shiman SL, Webster TF, Mora AM, Harris MH, Calafat AM (2015). Sociodemographic and perinatal predictors of early pregnancy per- and polyfluoroalkyl substance (PFAS) concentrations. Environ Sci Technol.

[11] Eick SM, Hom Thepaksorn EK, Izano MA, Cushing LJ, Wang Y, Smith SC (2020). Associations between prenatal maternal exposure to per- and polyfluoroalkyl substances (PFAS) and polybrominated diphenyl ethers (PBDEs) and birth outcomes among pregnant women in San Francisco. Environ Health.

[12] Vuong AM, Yolton K, Xie C, Dietrich KN, Braun JM, Webster GM (2019). Prenatal and childhood exposure to poly- and perfluoroalkyl substances (PFAS) and cognitive development in children at age 8 years. Environ Res.

[13] Fisher M, Arbuckle TE, Liang CL, Leblanc A, Gaudreau E, Foster WG (2016). Concentrations of persistent organic pollutants in maternal and cord blood from the maternal-infant research on environmental chemicals (MIREC) cohort study. Environ Health.

[14] Manzano-Salgado CB, Casas M, Lopez-Espinosa M-J, Ballester F, Basterrechea M, Grimalt JO (2015). Transfer of perfluoroalkyl substances from mother to fetus in a Spanish birth cohort. Environ Res.

[15] Wang Y, Han W, Wang C, Zhou Y, Shi R, Bonefeld-Jørgensen EC (2019). Efficiency of maternal-fetal transfer of perfluoroalkyl and polyfluoroalkyl substances. Environ Sci Pollut Res.

[16] Zhao L, Zhang Y, Zhu L, Ma X, Wang Y, Sun H (2017). Isomer-specific transplacental efficiencies of perfluoroalkyl substances in human whole blood. Environ Sci Technol Lett.

[17] Zheng P, Liu Y, An Q, Yang X, Yin S, Ma LQ (2022). Prenatal and postnatal exposure to emerging and legacy per-/polyfluoroalkyl substances: levels and transfer in maternal serum, cord serum, and breast milk. Sci Total Environ.

[18] Lorber M, Egeghy PP (2011). Simple intake and pharmacokinetic modeling to characterize exposure of Americans to perfluoroctanoic acid, PFOA. Environ Sci Technol.

[19] Oulhote Y, Steuerwald U, Debes F, Weihe P, Grandjean P (2016). Behavioral difficulties in 7-year old children in relation to developmental exposure to perfluorinated alkyl substances. Environ Int.

[20] Barry V, Winquist A, Steenland K (2013). Perfluorooctanoic acid (PFOA) exposures and incident cancers among adults living near a chemical plant. Environ Health Perspect.

[21] Winquist A, Steenland K (2014). Perfluorooctanoic acid exposure and thyroid disease in community and worker cohorts. Epidemiology..

[22] Melzer D, Rice N, Depledge MH, Henley WE, Galloway TS (2010). Association between serum perfluorooctanoic acid (PFOA) and thyroid disease in the U.S. National Health and Nutrition Examination Survey. Environ Health Perspect.

[23] Kim D-H, Kim U-J, Kim H-Y, Choi S-D, Oh J-E (2016). Perfluoroalkyl substances in serum from South Korean infants with congenital hypothyroidism and healthy infants—its relationship with thyroid hormones. Environ Res.

[24] Coperchini F, Awwad O, Rotondi M, Santini F, Imbriani M, Chiovato L. Thyroid disruption by perfluorooctane sulfonate (PFOS) and perfluorooctanoate (PFOA). J Endocrinol Invest. 2017;40:105–21. 10.1007/s40618-016-0572-z. 10.1007/s40618-016-0572-z 27837466

[25] Jugan M-L, Levi Y, Blondeau J-P (2010). Endocrine disruptors and thyroid hormone physiology. Biochem Pharm.

[26] Kim CS, Zhu X (2009). Lessons from mouse models of thyroid cancer. Thyroid.

[27] Franco AT, Malaguarnera R, Refetoff S, Liao X-H, Lundsmith E, Kimura S (2011). Thyrotrophin receptor signaling dependence of Braf-induced thyroid tumor initiation in mice. Proc Natl Acad Sci.

[28] Miller MD, Crofton KM, Rice DC, Zoeller RT (2009). Thyroid-disrupting chemicals: interpreting upstream biomarkers of adverse outcomes. Environ Health Perspect.

[29] Calsolaro V, Pasqualetti G, Niccolai F, Caraccio N, Monzani F. Thyroid disrupting chemicals. Int J Mol Sci. 2017;18:2583. 10.3390/ijms18122583 PMC5751186 29194390

[30] Kim MJ, Moon S, Oh BC, Jung D, Ji K, Choi K (2018). Association between perfluoroalkyl substances exposure and thyroid function in adults: a meta-analysis. PLoS ONE.

[31] Lee JE, Choi K (2017). Perfluoroalkyl substances exposure and thyroid hormones in humans: epidemiological observations and implications. Ann Pediatr Endocrinol Metab.

[32] Butenhoff JL, Chang S-C, Ehresman DJ, York RG (2009). Evaluation of potential reproductive and developmental toxicity of potassium perfluorohexanesulfonate in Sprague Dawley rats. Reprod Toxicol.

[33] Martin MT, Brennan RJ, Hu W, Ayanoglu E, Lau C, Ren H (2007). Toxicogenomic study of triazole fungicides and perfluoroalkyl acids in rat livers predicts toxicity and categorizes chemicals based on mechanisms of toxicity. Toxicol Sci.

[34] Yu W-G, Liu W, Jin Y-H (2009). Effects of perfluorooctane sulfonate on rat thyroid hormone biosynthesis and metabolism. Environ Toxicol Chem.

[35] Blake BE, Pinney SM, Hines EP, Fenton SE, Ferguson KK (2018). Associations between longitudinal serum perfluoroalkyl substance (PFAS) levels and measures of thyroid hormone, kidney function, and body mass index in the Fernald Community Cohort. Environ Pollut.

[36] Jobst KJ, Arora A, Pollitt KG, Sled JG (2020). Dried blood spots for the identification of bioaccumulating organic compounds: current challenges and future perspectives. Curr Opin Environ Sci Health.

[37] Hagstrom AL, Anastas P, Boissevain A, Borrel A, Deziel NC, Fenton SE (2021). Yale School of Public Health symposium: an overview of the challenges and opportunities associated with per- and polyfluoroalkyl substances (PFAS). Sci Total Environ.

[38] Petrick L, Edmands W, Schiffman C, Grigoryan H, Perttula K, Yano Y (2017). An untargeted metabolomics method for archived newborn dried blood spots in epidemiologic studies. Metabolomics.

[39] Petrick LM, Uppal K, Funk WE (2020). Metabolomics and adductomics of newborn bloodspots to retrospectively assess the early-life exposome. Curr Opin Pediatr.

[40] McClendon-Weary B, Putnick DL, Robinson S, Yeung E (2020). Little to give, much to gain—what can you do with a dried blood spot?. Curr Environ Heal Rep.

[41] Jacobson TA, Kler JS, Bae Y, Chen J, Ladror DT, Iyer R, et al. A state-of-the-science review and guide for measuring environmental exposure biomarkers in dried blood spots. J Expo Sci Environ Epidemiol. 2022;33:505–23. 10.1038/s41370-022-00460-7 PMC9375076 35963945

[42] Barr DB, Kannan K, Cui Y, Merrill L, Petrick LM, Meeker JD (2021). The use of dried blood spots for characterizing children’s exposure to organic environmental chemicals. Environ Res.

[43] Kato K, Wanigatunga AA, Needham LL, Calafat AM (2009). Analysis of blood spots for polyfluoroalkyl chemicals. Anal Chim Acta.

[44] Ma W, Kannan K, Wu Q, Bell EM, Druschel CM, Caggana M (2013). Analysis of polyfluoroalkyl substances and bisphenol A in dried blood spots by liquid chromatography tandem mass spectrometry. Anal Bioanal Chem.

[45] Spliethoff HM, Tao L, Shaver SM, Aldous KM, Pass KA, Kannan K (2008). Use of newborn screening program blood spots for exposure assessment: declining levels of perfluorinated compounds in New York State infants. Environ Sci Technol.

[46] Poothong S, Papadopoulou E, Lundanes E, Padilla-Sánchez JA, Thomsen C, Haug LS (2019). Dried blood spots for reliable biomonitoring of poly- and perfluoroalkyl substances (PFASs). Sci Total Environ.

[47] Koelmel JP, Lin EZ, Parry E, Stelben P, Rennie EE, Godri Pollitt KJ. Novel perfluoroalkyl substances (PFAS) discovered in whole blood using automated non-targeted analysis of dried blood spots. Sci Total Environ. 2023;883:163579. 10.1016/j.scitotenv.2023.163579 PMC10247435 37100129

[48] Lin EZ, Nason SL, Zhong A, Fortner J, Godri KJ (2023). Trace analysis of per- and polyfluorinated alkyl substances (PFAS) in dried blood spots—demonstration of reproducibility and comparability to venous blood samples. Sci Total Environ.

[49] Ghassabian A, Bell EM, Ma WL, Sundaram R, Kannan K, Buck Louis GM (2018). Concentrations of perfluoroalkyl substances and bisphenol A in newborn dried blood spots and the association with child behavior. Environ Pollut.

[50] Jones LE, Ghassabian A, Lawrence DA, Sundaram R, Yeung E, Kannan K (2022). Exposure to perfluoroalkyl substances and neonatal immunoglobulin profiles in the upstate KIDS study (2008–2010). Environ Pollut.

[51] Robinson SL, Zeng X, Guan W, Sundaram R, Mendola P, Putnick DL (2021). Perfluorooctanoic acid (PFOA) or perfluorooctane sulfonate (PFOS) and DNA methylation in newborn dried blood spots in the Upstate KIDS cohort. Environ Res.

[52] Yeung EH, Bell EM, Sundaram R, Ghassabian A, Ma W, Kannan K (2019). Examining endocrine disruptors measured in newborn dried blood spots and early childhood growth in a prospective cohort. Obesity..

[53] Gross RS, Ghassabian A, Vandyousefi S, Messito MJ, Gao C, Kannan K, et al. Persistent organic pollutants exposure in newborn dried blood spots and infant weight status: a case-control study of low-income Hispanic mother-infant pairs. Environ Pollut. 2020;267:115427. 10.1016/j.envpol.2020.115427 PMC7708683 33254620

[54] Rose SR, Brown RS (2006). Update of newborn screening and therapy for congenital hypothyroidism. Pediatrics.

[55] Kharrazi M, Pearl M, Yang J, DeLorenze GN, Bean CJ, Callaghan WM (2012). California very preterm birth study: design and characteristics of the population- and biospecimen bank-based nested case-control study. Paediatr Perinat Epidemiol.

[56] Hertzberg V, Mei J, Therrell BL (2010). Effect of laboratory practices on the incidence rate of congenital hypothyroidism. Pediatrics..

[57] Büyükgebiz A (2013). Newborn screening for congenital hypothyroidism. J Clin Res Pediatr Endocrinol.

[58] Waller DK, Anderson JL, Lorey F, Cunningham GC (2000). Risk factors for congenital hypothyroidism: an investigation of infant’s birth weight, ethnicity, and gender in California, 1990-1998. Teratology.

[59] Blackburn ST. Maternal, fetal, and neonatal physiology. 3rd ed. St. Louis: Elsevier; 2007.

[60] Huset CA, Barry KM (2018). Quantitative determination of perfluoroalkyl substances (PFAS) in soil, water, and home garden produce. MethodsX..

[61] Munoz G, Ray P, Mejia-Avendaño S, Duy SV, Do DT, Liu J (2018). Optimization of extraction methods for comprehensive profiling of perfluoroalkyl and polyfluoroalkyl substances in firefighting foam impacted soils. Anal Chim Acta.

[62] Nason SL, Koelmel J, Zuverza-Mena N, Stanley C, Tamez C, Bowden JA (2021). Software comparison for nontargeted analysis of PFAS in AFFF-contaminated soil. J Am Soc Mass Spectrom.

[63] Adam BW, Alexander JR, Smith SJ, Chace DH, Loeber JG, Elvers LH (2000). Recoveries of phenylalanine from two sets of dried-blood-spot reference materials: prediction from hematocrit, spot volume, and paper matrix. Clin Chem.

[64] Kadjo AF, Stamos BN, Shelor CP, Berg JM, Blount BC, Dasgupta PK (2016). Evaluation of amount of blood in dry blood spots: ring-disk electrode conductometry. Anal Chem.

[65] Lee EC, Whitehead AL, Jacques RM, Julious SA. The statistical interpretation of pilot trials: should significance thresholds be reconsidered? BMC Med Res Methodol. 2014;14:41. 10.1186/1471-2288-14-41 PMC3994566 24650044

[66] Chang SC, Das K, Ehresman DJ, Ellefson ME, Gorman GS, Hart JA (2008). Comparative pharmacokinetics of perfluorobutyrate in rats, mice, monkeys, and humans and relevance to human exposure via drinking water. Toxicol Sci.

[67] Haug LS, Thomsen C, Becher G (2009). Time trends and the influence of age and gender on serum concentrations of perfluorinated compounds in archived human samples. Environ Sci Technol.

[68] Hurley S, Goldberg D, Wang M, Park J-S, Petreas M, Bernstein L (2018). Time trends in per- and polyfluoroalkyl substances (PFASs) in California women: declining serum levels, 2011–2015. Environ Sci Technol.

[69] Land M, De Wit CA, Bignert A, Cousins IT, Herzke D, Johansson JH (2018). What is the effect of phasing out long-chain per-and polyfluoroalkyl substances on the concentrations of perfluoroalkyl acids and their precursors in the environment? A systematic review. Environ Evid.

[70] Schecter A, Malik-Bass N, Calafat AM, Kato K, Colacino JA, Gent TL (2012). Polyfluoroalkyl compounds in Texas children from birth through 12 years of age. Environ Heal Perspect.

[71] Ye X, Kato K, Wong L-Y, Jia T, Kalathil A, Latremouille J (2018). Per- and polyfluoroalkyl substances in sera from children 3 to 11 years of age participating in the National Health and Nutrition Examination Survey 2013–2014. Int J Hyg Environ Health.

[72] Calafat AM, Wong LY, Kuklenyik Z, Reidy JA, Needham LL (2007). Polyfluoroalkyl chemicals in the U.S. population: data from the national health and nutrition examination survey (NHANES) 2003-2004 and comparisons with NHANES 1999-2000. Environ Health Perspect.

[73] Preston EV, Webster TF, Claus Henn B, McClean MD, Gennings C, Oken E (2020). Prenatal exposure to per- and polyfluoroalkyl substances and maternal and neonatal thyroid function in the Project Viva Cohort: a mixtures approach. Environ Int.

[74] Berg V, Nøst TH, Pettersen RD, Hansen S, Veyhe A-S, Jorde R (2017). Persistent organic pollutants and the association with maternal and infant thyroid homeostasis: a multipollutant assessment. Environ Health Perspect.

[75] Preston EV, Webster TF, Oken E, Claus Henn B, McClean MD, Rifas-Shiman SL (2018). Maternal plasma per- and polyfluoroalkyl substance concentrations in early pregnancy and maternal and neonatal thyroid function in a prospective birth cohort: project viva (USA). Environ Health Perspect.

[76] De Cock M, De Boer MR, Lamoree M, Legler J, Van De Bor M (2014). Prenatal exposure to endocrine disrupting chemicals in relation to thyroid hormone levels in infants—a Dutch prospective cohort study. Environ Health.

[77] Dufour P, Pirard C, Seghaye MC, Charlier C (2018). Association between organohalogenated pollutants in cord blood and thyroid function in newborns and mothers from Belgian population. Environ Pollut.

[78] Ballesteros V, Costa O, Iniguez C, Fletcher T, Ballester F, Lopez-Espinosa MJ (2017). Exposure to perfluoroalkyl substances and thyroid function in pregnant women and children: a systematic review of epidemiologic studies. Environ Int.

