# Supplementary material for: Per- and polyfluoroalkyl substances (PFAS) and thyroid hormone measurements in dried blood spots and neonatal characteristics: a pilot study
Source: J Expo Sci Environ Epidemiol. 2023 Sep 20;33(5):737–47. doi: 10.1038/s41370-023-00603-4 (PMC10541328; doi:10.1038/s41370-023-00603-4)
Supplement: Supplementary file 1 — Supplementary Material [file 41370_2023_603_MOESM1_ESM.docx]

**Supplementary Material**

Per- and Polyfluoroalkyl Substances (PFAS) and Thyroid Hormone Measurements in Dried Blood Spots and Neonatal Characteristics: A Pilot Study

**Authors:** Ana K. Rosen Vollmar, Elizabeth Z. Lin, Sara L. Nason, Katerina Santiago, Caroline H. Johnson, Xiaomei Ma, Krystal J. Godri Pollitt, Nicole C. Deziel

**Table of contents**

**Table S1.** Per- and polyfluoroalkyl substances (PFAS) quantified in newborn dried blood spots (DBS)

**Table S2.** Percent difference between PFAS in paired dried blood spots (DBS) and card blanks calculated using Equation S1

**Table S3.** PFAS concentrations (pg/g) in 18 neonatal dried blood spots stratified by infant sex and race/ethnicity

**Table S4.** Participant characteristics of 18 infants stratified by the number of different PFAS detected in individual dried blood spot samples

**Supplemental Material:** Example calculation for PFAS concentration in units of pg/g dried blood

| **Table S1.** Per- and polyfluoroalkyl substances (PFAS)  quantified in newborn dried blood spots (DBS) | |
| --- | --- |
| PFAS Name | Abbreviation |
| 4:2 fluorotelomer sulfonic acid | 4:2 FTS |
| 6:2 fluorotelomer sulfonic acid | 6:2 FTS |
| 8:2 fluorotelomer sulfonic acid | 8:2 FTS |
| Perfluorooctane sulfonamide | PFOSA |
| Perfluorobutane sulfonic acid | PFBS |
| Perfluoropentane sulfonic acid | PFPeS |
| Perfluorohexane sulfonic acid | PFHxS |
| Perfluoroheptane sulfonic acid | PFHpS |
| Perfluorooctane sulfonic acid | PFOS |
| Perfluorononane sulfonic acid | PFNS |
| Perfluorodecane sulfonic acid | PFDS |
| Perfluorobutanoic acid | PFBA |
| Perfluoropentanoic acid | PFPeA |
| Perfluorohexanoic acid | PFHxA |
| Perfluoroheptanoic acid | PFHpA |
| Perfluorooctanoic acid | PFOA |
| Perfluorononanoic acid | PFNA |
| Perfluorodecanoic acid | PFDA |
| Perfluoroundecanoic acid | PFUnA |
| Perfluorododecanoic acid | PFDoA |
| Perfluorotridecanoic acid | PFTrDA |
| Perfluorotetradecanoic acid | PFTeDA |

| **Table S2.** Percent difference between PFAS in paired dried blood spots (DBS) and card blanks calculated using Equation S1 | | | | |
| --- | --- | --- | --- | --- |
|  | Minimum | Median | Average | Maximum |
| 6:2 FTS | -168.9 | 2.6 | -9.8 | 88.6 |
| PFBA | 63.0 | 80.0 | 78.6 | 91.4 |
| PFBS | 55.6 | 87.2 | 81.1 | 98.5 |
| PFHpA | 48.6 | 48.6 | 48.6 | 48.6 |
| PFHpS | 81.2 | 91.0 | 89.4 | 94.4 |
| PFHxS | 93.5 | 97.4 | 96.9 | 98.5 |
| PFOA | 52.1 | 86.6 | 83.9 | 95.7 |
| PFOS | 92.8 | 98.1 | 97.6 | 99.0 |
| PFOSA | 71.5 | 92.9 | 90.0 | 98.4 |

Notes: Units are percent difference, calculated using Equation S1:

**Equation S1:** $\text{\% Difference}=\frac{\text{C}_{\text{DBScard}}\text{ -}\text{ C}_{\text{BLKcard}}}{\text{C}_{\text{DBScard}}}\text{×100\%}$

*C_DBScard_:* PFAS concentration in the dried blood spot by the card component, ng_PFAS_/g_card_

*C_BLKcard_:* PFAS concentration in the card blank paper, ng_PFAS_/g_card_

Abbreviations: PFAS, per- and polyfluoroalkyl substances; 6:2 FTS, 6:2 fluorotelomer sulfonic acid; PFBA perfluorobutanoic acid; PFBS, perfluorobutane sulfonic acid; PFHpA, perfluoroheptanoic acid; PFHpS perfluoroheptane sulfonic acid; PFHxS, perfluorohexane sulfonic acid; PFOA, perfluorooctanoic acid; PFOS, perfluorooctane sulfonic acid; PFOSA perfluorooctane sulfonamide.

| **Table S3.** PFAS concentrations (pg/g) in 18 neonatal dried blood spots stratified by infant sex and race/ethnicity | | | | | | | | |
| --- | --- | --- | --- | --- | --- | --- | --- | --- |
| Participant or sample characteristic | PFOA,  median (IQR) | PFOS,  median (IQR) | PFOSA, median (IQR) | PFBA,  median (IQR) | PFBS,  median (IQR) | PFHpA, median (IQR) | PFHpS, median (IQR) | PFHxS, median (IQR) |
| Full cohort (n=18) | 848.2 (<LOD, 1503.7) | 739.1 (<LOD, 2705.5) | 519.5 (<LOD, 715.3) | <LOD | <LOD (<LOD, 941.6) | <LOD | <LOD | <LOD (<LOD, 184.6) |
| Samples >LOD, *n* (%) | 10 (56%) | 9 (50%) | 12 (67%) | 4 (22%) | 8 (44%) | 1 (6%) | 4 (22%) | 5 (28%) |
| Infant sex |  |  |  |  |  |  |  |  |
| Male (n=9) | <LOD (<LOD, 872.8) | <LOD (<LOD, 2446.0) | 687.3 (<LOD, 758.4) | <LOD | <LOD (<LOD, 549.0) | <LOD | <LOD | <LOD |
| Female (n=9) | 1008.8 (<LOD, 1839.8) | 1478.2 (<LOD, 4891.3) | 407.7 (<LOD, 704.6) | <LOD | <LOD (<LOD, 941.6) | <LOD | <LOD (<LOD, 127.4) | <LOD (<LOD, 184.6) |
| Race/Ethnicity |  |  |  |  |  |  |  |  |
| White (n=6) | <LOD (<LOD, 2261.1) | 2965.2 (<LOD, 4891.3) | 554.2 (<LOD, 1083.3) | <LOD (<LOD, 2636.2) | <LOD (<LOD, 1536.5) | <LOD | <LOD (<LOD, 127.4) | 254.5 (<LOD, 891.6) |
| Hispanic (n=3), Black (n=2), Asian (n=3) | 990.5 (<LOD, 1340.1) | <LOD (<LOD, 1824.2) | 70.6 (<LOD, 556.2) | <LOD | 189.1 (<LOD, 527.7) | <LOD | <LOD (<LOD, 16.4) | <LOD |
| Unknown (n=4) | 848.2 (411.8, 1304.9) | 1223.0 (<LOD, 2575.8) | 701.3 (656.3, 843.3) | <LOD (<LOD, 2028.8) | 547.7 (<LOD, 2593.3) | <LOD | <LOD | <LOD (<LOD, 120.8) |

Abbreviations: PFAS, per- and polyfluoroalkyl substances; PFOA, perfluorooctanoic acid; PFOS, perfluorooctane sulfonic acid; PFOSA perfluorooctane sulfonamide; PFBA perfluorobutanoic acid; PFBS, perfluorobutane sulfonic acid; PFHpA, perfluoroheptanoic acid; PFHpS perfluoroheptane sulfonic acid; PFHxS, perfluorohexane sulfonic acid; IQR, interquartile range; LOD, limit of detection.

| **Table S4.** Participant characteristics of 18 infants stratified by the number of different PFAS detected in individual dried blood spot samples | | |
| --- | --- | --- |
| Participant characteristics | Participants with 0-2 PFAS detected, *n* ^a^ | Participants with 3-6 PFAS detected, *n* ^a^ |
| Full cohort | 8 | 10 |
| Sex |  |  |
| Male | 5 | 4 |
| Female | 3 | 6 |
| Race/ethnicity |  |  |
| White | 3 | 3 |
| Hispanic (n=3), Black (n=2), Asian (n=3) | 4 | 4 |
| Unknown | 1 | 3 |

Abbreviations: PFAS, per- and polyfluoroalkyl substances.

^a^ The median number of PFAS detected in cohort samples was 3.

**Supplemental Material:** Example calculation for PFAS concentration in units of pg/g dried blood

**Samples:**

80 mg card blank (area: 500 mm^2^)

110 mg DBS (area: 500 mm2) – contains 30 mg dried blood and 80 mg card (subtract card blank mass for an equivalent area sample)

Both are prepared into 0.1 mL of extract

**Measurements:**

Card blank: 100 pg/mL PFOS

DBS: 500 pg/mL PFOS

**Blank Comparison and subtraction**:

The card blank contains 0.125 pg PFOS per mg card (multiply measurement by 0.1 mL extract, divide by 80 mg card)

The DBS contains 0.625 pg PFOS per mg card (multiply measurement by 0.1 mL extract, divide by 80 mg card)

The card blank is > 20% lower than the DBS measurement, so the data is not excluded.

0.625 – 0.125 = 0.5 pg PFOS per mg card

40 pg total in the DBS sample that are not present in the card blank (multiply by 80 mg card mass)

**Conversion to ng/g dried blood:**

40 pg PFOS total ÷ 30 mg dried blood = 1.33 pg/mg

x1000 to convert to pg/g = ***1330 pg/g*** ***PFOS in dried blood*** (reported value)
